# 

**Published:** 1879-03

**Authors:** 


					﻿MARCH, 1879.
TWENTY-SIXTH YEAR—No 303.
ITAJJA
Journal of Health.
PUBLISHED AT 141 EIGHTH STREET,
(Near Broadway),	NEW YORK.
E. H. GIBBS, A.M., M.D., Editor.
AGENTS FOR THE TRADE :
AMERICAN NEWS COMPANY AND NEW YORK NEWS COMPANY
Terms, $1.50 a Year, or $1.00 for Eight Months. Postage paid.
SINGLE NUMBER, IB CENTS.
Couch & Johnston, Printers, 11 Frankfort Street, NewYork.
				

